# First Detection of West Nile Virus Lineage 2 in *Culex pipiens* Vectors in Croatia

**DOI:** 10.3390/pathogens13121131

**Published:** 2024-12-21

**Authors:** Goran Vignjević, Nataša Bušić, Nataša Turić, Zsaklin Varga, Brigitta Zana, Ágota Ábrahám, Kornélia Kurucz, Ivana Vrućina, Enrih Merdić

**Affiliations:** 1 Department of Biology, University Josip Juraj Strossmayer of Osijek, 31000 Osijek, Croatia; goran@biologija.unios.hr (G.V.); ivrucina@biologija.unios.hr (I.V.); enrih@biologija.unios.hr (E.M.); 2 Teaching Institute of Public Health of Osijek-Baranja County, 31000 Osijek, Croatia; 3 National Laboratory of Virology, Szentagothai Research Centre, University of Pécs, 7600 Pécs, Hungary; v.zsaklin95@gmail.com (Z.V.); brigitta.zana@gmail.com (B.Z.); agotaabraham@gmail.com (Á.Á.); kornelia.kurucz@gmail.com (K.K.); 4 Institute of Biology, Faculty of Sciences, University of Pécs, 7600 Pécs, Hungary

**Keywords:** VBD, tiger mosquito, *Aedes albopictus*, common house mosquito, *Culex pipiens*, arbovirus, surveillance, One Health

## Abstract

The West Nile virus (WNV) has recently become more widespread, posing a threat to both human and animal health. In Western Europe, most outbreaks have been caused by WNV lineage 1, while in Eastern Europe, WNV lineage 2 has led to human and bird mortality. The ability to appropriately manage this threat is dependent on integrated surveillance and early detection. This study aimed to quantify the prevalence of WNV infection in mosquitoes and to identify the circulating viral lineage in eastern Croatia. Mosquito traps were set up in rural and urban areas during the 2021–2023 seasons, and the collected specimens were identified morphologically. Mosquito species *Culex pipiens* and *Aedes albopictus* were tested for Flaviviruses using conventional PCR in a heminested system. The positive samples were then subjected to a specific real-time PCR designed to detect WNV. A total of 385 mosquito pools were tested, and positive pools were found in samples from Osijek-Baranja and Vukovar-Srijem, both of which contained *Cx. pipiens* mosquitoes. Sequencing of amplicons revealed WNV lineage 2 partial NS5 gene sequences. Phylogenetic analysis suggests the Hungarian origin of strain, which complements birds’ migratory routes. These findings indicate the first detection of WNV in mosquitoes in Croatia. This suggests that human cases in this region are likely due to infections with lineage 2 transmitted by local *Culex* mosquitoes.

## 1. Introduction

The West Nile virus (WNV) is a member of the genus *Flavivirus* and belongs to the Japanese encephalitis serocomplex of the Flaviviridae family [1]. Beyond its enzootic cycle, WNV can infect humans, horses, and other mammals, causing severe diseases or mortality [2]. WNV was first isolated in Uganda, in the West Nile province, in 1937 [3]. Since then, it has become a major public health concern during the last two decades, spreading from Africa to the southern parts of Europe, Asia, and Australasia by migratory birds. Indeed, studies have shown that migratory routes often overlap with regions where the virus is endemic, contributing to the spread during migration seasons [4,5]. Birds such as corvids, sparrows, and raptors can sustain high viral loads without exhibiting symptoms, facilitating the maintenance and amplification of the virus in nature [6]. The largest epidemics of WNV have occurred in Italy, Greece, Israel, Romania, Russia, and the USA [2,7]. In many European countries, WNV infections have been occurring continuously since 2008 [8,9]. In Italy, an important epidemiological area with significant WNV activity, the number of human cases has fluctuated significantly over the years. The first major outbreak occurred in 2008, and several confirmed cases of neuroinvasive disease were reported. Subsequent years saw continued reports, with notable outbreaks in 2012 and 2018, which were particularly severe with over 200 reported cases, highlighting the virus’s persistence and potential for outbreaks [10].

In Croatia, acute WNV infections, as well as seropositivity, have been detected in humans, horses, birds, and poultry. Serological evidence from the 1970s suggests an early circulation of WNV in Croatia, although the first clinical cases emerged in 2012 [11]. Epidemics of WNV, as well as sporadic infections, were continuously recorded in the counties of continental Croatia from 2012 to 2018 [12]. During August and September 2012, seven cases of West Nile neuroinvasive disease (WNND) were identified in humans in three northeastern counties of Croatia. Four cases were recorded in Osijek-Baranja County, two in Brod-Posavina, and one in Vukovar-Srijem County [13]. From 2014 to 2016, isolated cases were reported, followed by a minor eight-case epidemic in 2017. The largest documented outbreak occurred in 2018, with 54 WNND cases and seven WNV fever cases in 11 Croatian counties. After a three-year hiatus in reported cases, six infections were documented across three counties in 2022 [14].

The first acute asymptomatic infections in horses were recorded in 2001 and 2002 in eight Croatian counties [15]. Between 2010 and 2011, WNV was highly present in Croatia, spreading from east to west, with the highest rates in eastern regions and westernmost Istria) [12]. Eastern regions show high seroprevalence rates, indicating they are part of the wider European endemic area of WNV [16]. Acutely infected animals emerged in seven counties during the 2018 Croatian epidemic, and animals that tested positive for IgG were detected in every continental county. The areas with the largest number of human cases correlated with the highest seropositivity [12], which was also the case in the neighboring region [17]. The exceptionally warm and humid conditions in Croatia’s continental region during 2018 may have facilitated an earlier onset of the West Nile Virus transmission season [18]. In continental counties, high WNV seropositivity in horses continued in 2019 and 2020. Since 2012, infections have been continuously recorded, but the first clinical infection with neurological signs in horses was registered in 2022 [19].

WNV RNA was detected in the brain tissues of 35 deceased wild birds during the 2018 WNV epidemic. As WNV sentinels, chickens and turkeys were observed, and the first case of poultry infections was reported in 2013. WNV circulation was observed in Croatia between 2013 and 2020, with seropositivity primarily in continental counties [14]. Findings of these seroprevalence rates, although they are not a direct confirmation of WNV infection, highlighted the continental, and especially eastern part of Croatia as areas with the most probable transmission of WNV to humans and animals.

*Culex pipiens* s.l. is the main WNV vector in Europe due to its competence and abundance. Although the virus was also found in other mosquitoes within the genus *Culex*, transmission competence was experimentally proven for *Cx. torrentium* and the invasive species *Aedes albopictus* [8,20]. Both *Cx. pipiens* s.l. and *Ae. albopictus* are widespread in Croatia, especially *Ae. albopictus*, invading all Croatian counties in the last 20 years and becoming dominant in urban areas.

WNV consists of nine defined lineages [21], of which lines 1 and 2 are associated with human diseases [22,23]. Over the past 50 years, lineage 1 caused WNV epidemics in the Mediterranean [24], and it was linked to severe reports in horses, birds, and humans, suggesting a higher pathogenicity than lineage 2. Lineage 2 was first detected in Southeast Hungary in 2004 and quickly expanded to neighboring countries [25,26,27]. Recent outbreaks indicate that WNND can be deadly and severe in both lineages [28,29].

Besides WNV, further arthropod-borne viruses transmitted by mosquitoes have also emerged recently in Croatia. The first evidence of flaviviruses in mosquitoes in Croatia was recorded in 2016 in Zagreb, namely the Usutu virus RNA in pooled samples of the invasive mosquito species *Ae. albopictus*. The detection of this virus in Zagreb during the years of research shows that the virus is continuously present in the researched area and that northwestern Croatia has become an endemic area for USUV [30]. Concerning the chikungunya virus, a seroprevalence study conducted among the inhabitants of Mediterranean Croatia (2011 to 2012) showed a seroprevalence rate of 0.9% (0.5 to 1.8%), which is evidence of chikungunya virus circulation [31]. Although autochthonous chikungunya and Zika virus infections have not been recorded up to date, sporadic imported cases have been continuously reported since 2016 [32].

Early detection of pathogens in vector or reservoir species is crucial for managing vector-borne diseases before human infections and epidemics. Even though there are frequent human cases of WNND in Croatia, the presence of WNV has not been recorded in mosquitoes [12,13,30,33]. In 2011, the Croatian Ministry of Agriculture, Fisheries, and Rural Development introduced a passive flavivirus surveillance program, which led to ongoing seroprevalence studies in sentinel horses and poultry. Additionally, from 2017 to 2021, the CRONEUROARBO project investigated the prevalence and molecular epidemiology of emerging and reemerging neuroinvasive arboviral infections. This project involved the detection of arboviruses in humans, sentinel animals (horses, birds, and poultry), and vectors (mosquitoes and ticks) across continental Croatia [12]. These projects resulted in reports of WNV infections in humans, horses, birds, and poultry but without infections in mosquitoes. The possible reasons why WNV has not been previously detected in mosquitoes are yet to be determined. Whether it was sampling strategy, small sample, operator experience, laboratory techniques, cold chain handling, or a combination of these, it is out of our reach. Therefore, this study aimed to assess the presence of WNV in mosquitoes and to identify the circulating viral lineage in eastern Croatia, an area that reports the highest frequency of human cases of WNND.

## 2. Materials and Methods

### 2.1. Study Area and Mosquito Sampling

Mosquito traps—dry ice baited CDC traps (Bioquip, Compton, CA, USA)—and BG sentinel traps (Biogents AG, Regensburg, Germany) were placed in rural and urban sites across the three different counties in eastern Croatia. Samples in Osijek-Baranja county were obtained in 2021, from Požega-Slavonia county in 2022, and Vukovar-Srijem county in 2023. Traps operated monthly throughout the season, from May to September, at selected sites. CDC traps were set in green areas in the evening and collected the next morning, while BG sentinel traps were predominantly placed in backyards and left to operate for one week with a daily exchange of collection nets. Collected specimens were transported in boxes with dry ice, and cold plates and standard keys were used to morphologically identify mosquitoes at the species level. Then, *Cx. pipiens* and *Ae. albopictus* specimens were pooled by collection date, location, and species in a total of 385 pools with up to 25 individuals and stored at −80 °C. Samples were processed for the pathogen screening one to two weeks after sampling.

### 2.2. Nucleic Acid Extraction

For *Flavivirus* screening and identification in *Cx. pipiens* and *Ae. albopictus* mosquitoes, the sample processing followed the protocol published by Varga et al. [34] with changes in usage of the qRT-PCR Kit—Luna Universal Probe One-Step RT-qPCR Kit (New England BioLabs Inc., Ipswich, MA, USA) was used instead of the Brilliant III Ultra-Fast QPCR Master Mix (Agilent Technologies, Santa Clara, CA, USA)—and the method of sequencing (Oxford Nanopore amplicon sequencing was used instead of Illumina); these procedures were conducted under laboratory conditions. Prior to molecular analyses, mosquito pools were disrupted in 500 μL of phosphate-buffered saline (PBS) using glass beads and the Qiagen TissueLyser II machine (Qiagen GmbH, Hilden, Germany) instead of plastic sticks. The homogenization conditions were as follows: 30 Hz for 5 min. After homogenization, the samples were spun to remove a sufficient amount of supernatant. Nucleic acid extraction was performed based on the Beckman Coulter RNAdvance Viral XP (RNAdvance Viral XP 1.5 mL Tube Protocol).

### 2.3. General Flavivirus Detection with Conventional Heminested PCR and Sanger Sequencing

Mosquito pools (containing *Cx. pipiens* and *Ae. albopictus*) were tested for Flaviviruses by conventional PCR in a heminested system with previously published primers [35], amplifying a 250 bp long fragment in the NS5 gene in Flaviviruses (Table 1). With this system, the detection of approximately 2 × 10^2^ infectious doses/ml^−1^ is possible [35]. The 1st round of heminested PCR was made with the Qiagen OneStep RT-PCR Kit (Qiagen GmbH, Germany), with the following thermocycling conditions: 50 °C for 30 min for reverse transcription; 95 °C for 15 min for denaturation; 30 cycles of 94 °C for 1 min, 53 °C for 1 min, and 72 °C for 1 min for amplification; and 72 °C for 7 min for the final extension. The 2nd round of heminested PCR was made with GoTaq DNA Polymerase (Promega, Madison, WI, USA). The steps of the thermocycling program were 95 °C for 2 min; 35 cycles of 94 °C for 30 s, 54 °C for 1 min, and 72 °C for 1 min for amplification; and 72 °C for 5 min for the final extension. After PCR, gel electrophoresis was performed with 1.5% agarose gel and SYBR Safe DNA gel dye (Thermo Fisher Scientific, Carlsbad, CA, USA) for amplicon visualization. Then, PCR products were purified from the gel using the NEBMonarch PCR and DNA Clean-up Kit (New England Biolabs, USA), according to the manufacturer’s instructions. For the identification of flaviviruses, Sanger sequencing was processed by Microsynth AG (Balgach, Switzerland).

### 2.4. West-Nile Virus Detection with Real-Time PCR

Following gel electrophoresis, the *Flavivirus*-positive samples were further tested with a WNV-specific real-time PCR designed to detect the RNA of WNV. The PCR was performed with the Luna Universal Probe One-Step RT-qPCR Kit (New England BioLabs Inc., USA) and with previously published primers and probe [36] (Table 1), under the following conditions: 55 °C for 10 min reverse transcription; 95 °C for 1 min for initial denaturation, and 40 cycles of 95 °C for 10 s and 60 °C for 30 sec for amplification. With this TaqMan WNV quantitative assay, WNV RNA can be reliably detected at concentrations of up to 10 to 30 copies/mL [36]. The procedure was performed on the MyGo^®^ Mini S Real-Time PCR machine (IT-IS Life Science Ltd., Dublin, Ireland). After qRT-PCR, Auto Quantification (absolute and relative quantification, using model-based Cq calling) was used in the MyGo Mini Software v3.5.6. (IT-IS Life Science Ltd., Dublin, Ireland).

### 2.5. West-Nile Virus Sequencing and Phylogenetic Analysis

To obtain the complete genome, we performed WNV Lineage 2 specific amplicon-based sequencing on the Oxford Nanopore platform with primers previously published [37]. From the previously extracted RNA, cDNA was synthesized with the SuperScript™ IV First-Strand Synthesis System (Invitrogen, Waltham, MA, USA) using random hexamers following the manufacturer’s protocol. Two separate primer pools were generated, according to Diagne et al. [37]. Multiplex PCR was generated from cDNA with the primer pools using the Q5^®^ Hot Start High-Fidelity DNA Polymerase (New England Biolabs, USA) with thermocycling conditions suggested by the manufacturer. PCR amplicons were cleaned up with the AMPure XP Reagent (Beckman Coulter, Brea, CA, USA) and were quantified using the Qubit dsDNA HS Assay Kit (Invitrogen, Waltham, MA, USA) on a Qubit 4 fluorometer (Invitrogen, USA). MinIon libraries were performed with the Ligation Sequencing kit SQK-LSK-110 and Native Barcoding Kit EXP-NBD196 (ONT). For end repair and dA tailing of amplicons, the NEBNext Ultra II End Repair/dA-Tailing Module (New England Biolabs, USA) was used. Ligation of barcodes was performed with the NEBNext Ultra II Ligation Module (New England Biolabs, USA). Adaptors were ligated to the barcoded pools with the NEBNext Quick Ligation Module (New England Biolabs, USA). After adaptor ligation, libraries were cleaned up with Ampure XP beads (Beckman Coulter, USA) using 75% ethanol, except for the last cleanup where Small Fragment Buffer (ONT) was applied. The cleaned library was quantified using a Qubit dsDNA HS Assay Kit (Invitrogen, USA) on a Qubit 4 fluorometer (Invitrogen, USA). The final library was sequenced on an R9.4.1 (FLO-MIN106D) flow cell using an Mk1B MinIon device (ONT). Base-calling of raw data was performed by Guppy (ONT Guppy v6.3.2) using the super accuracy base-calling algorithm (dna_r9.4.1_450bps_sup config file). Demultiplexing and trimming of barcodes were performed with Guppy as well, using the default parameters of the ’guppy_barcoder’ runcode. Quality filtering and the trimming of overlapping regions of amplicons were conducted with the NanoFilt software (v2.8.0). Additional sequence manipulations were performed with the Geneious Prime^®^ 2024.0.5 software.

The maximum likelihood phylogenetic tree was constructed in the IQ-TREE software package v1.6.12. [38]. For phylogenetic analysis, we used the nearly complete amino acid sequence of the viral polyprotein. For the selection of the best-fit model, we used the ModelFinder implemented ultra-fast model selection feature [39]. The tree was generated according to the suggested best-fit model HIVb + F + I [40]. During analysis, we used the Bootstrap resampling method with 1000 replicates; the bootstrap threshold was 50%, and we considered nodes robustly supported with above 70% of bootstrap value. The constructed tree was visualized and edited in the iTOL online tool [41].

## 3. Results

**Figure 1:**
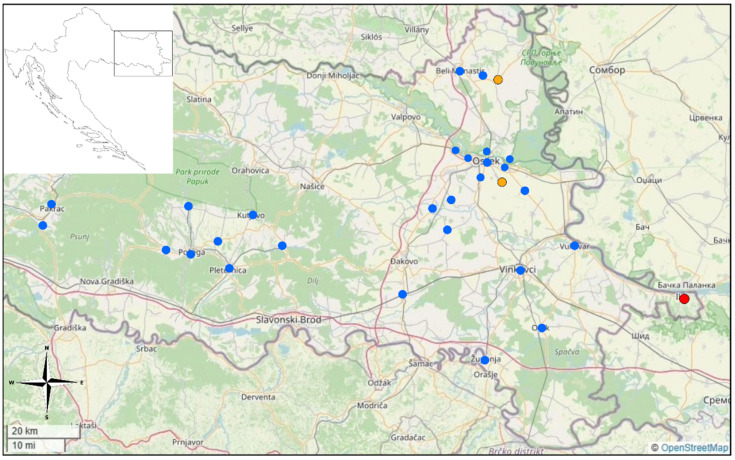
Map of the studied area of three counties in eastern Croatia. Orange dots are positive sites for *Flavivirus*, red dots are positive sites for WNV, and blue dots are negative sites.

The sampled mosquitoes belonged to 14 species. Of these, 86.4% of mosquitoes were identified as *Aedes vexans*, 7.9% as *Cx. pipiens* complex, 3.7% as *Ochlerotatus sticticus*, 0.7% as *Cx. modestus*, and 0.4% as *Ae. albopictus*. Lower percentages represented other species. From the sampled mosquitoes, all the collected *Cx. pipiens* and *Ae. albopictus* specimens were included in the analysis (pooled by date, location, and species in a total of 385 pools). From Osijek-Baranya county, 66 pools were made. Out of those, three pools were positive for flaviviruses containing *Cx. pipiens* mosquitoes. These samples originated from the villages Tenja and Kneževi Vinogradi (Figure 1) and were collected on 8 June and 1 July 2021, respectively. Due to the low viral load of the virus RNA, the virus nor its lineage could be detected in this case. From Vukovar-Srijem county, a total of 196 mosquito pools were processed, which resulted in one positive sample for the presence of *Flavivirus* containing *Cx. pipiens* in mosquito specimens collected on 27 July 2023 in the town center of Ilok (Figure 1). Based on the Sanger sequencing and BLAST (Basic Local Alignment Search Tool) search in the GenBank database, this sample showed 98% identity with West Nile virus isolate (genotype 2) originating from a human sample collected in August 2021 in Hungary (GenBank Acc.No.: OP179287.1).

The positive results of conventional PCR for Flaviviruses were confirmed by WNV-specific qRT-PCR, and both Sanger sequencing and real-time PCR showed positivity in the same samples.

In Požega-Slavonia county, 123 pools were tested, but no samples were positive for Flaviviruses.

**Figure 2:**
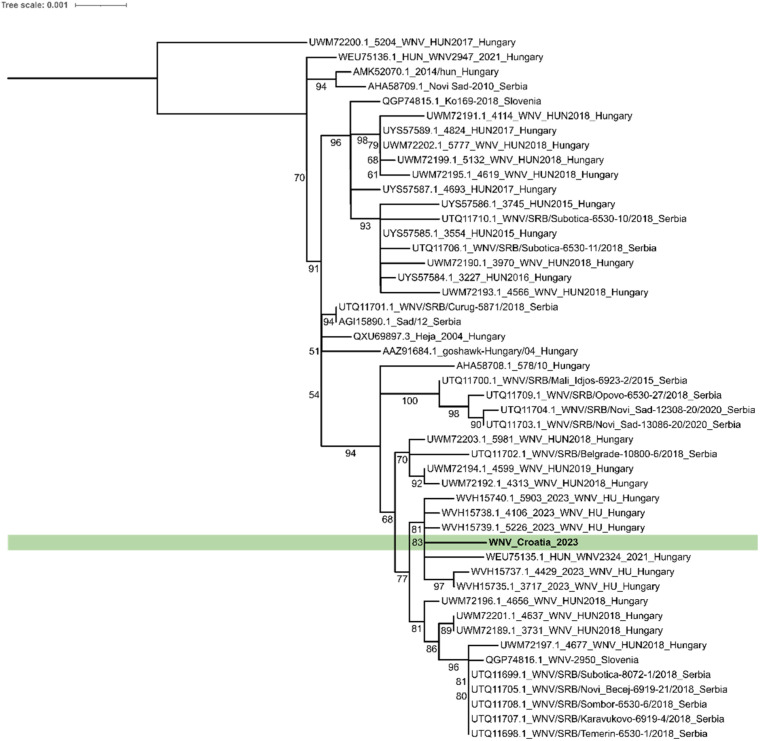
Maximum likelihood phylogenetic tree of the Croatian WNV isolate (GenBank database Acc.No.: PQ468649) and WNV isolates from neighboring countries. The tree was constructed based on the amino acid sequences of the complete polyprotein region of the viruses. All sequences belong to the Lineage 2 genetic variant. Sequences are indicated by their GenBank accession number, strain name, and country of origin. The sequence related to this study is highlighted with a light green background. The scale bar indicates the mean number of amino acid substitutions per site. The tree was created according to the best-fit model HIBb + F + I with a bootstrap resampling method with 1000 replicates.

During amplicon sequencing, the quality cutoff of reads was Q10, and from the obtained 211,367 reads, 211,258 (99.9%) reads fell into this category. Sequence coverage was accepted with a minimum threshold of 20-fold. The average coverage was 7903.8 reads per amplicon. As a result of amplicon sequencing and bioinformatic analysis, nearly the complete coding sequence was obtained from the WNV-positive mosquito homogenate. To determine the phylogenetic relationship between our WNV sequence and sequences from neighboring countries, a maximum-likelihood phylogenetic tree was constructed. The results of the phylogenetic analysis indicate that the strain identified in this study clusters within a distinct clade alongside Hungarian West Nile Virus strains isolated in 2023, implying a potential Hungarian origin for the virus (Figure 2).

## 4. Discussion

All arboviruses circulate among wild animals and can cause disease after incidental transmission to humans and domestic animals. Many pathogens that have become important in animal and human health are actually arboviruses that have developed new mechanisms of adaptation. Among these, WNV has dramatically expanded its geographic range in the past twenty years. Increases in global commerce, climate change, ecological factors, and the emergence of novel viral genotypes likely play significant roles in the emergence of this virus [42]. Additionally, many foreigners working in Europe may represent potential imported cases after traveling to areas with autochthonous arboviral transmission. Surveillance of arboviruses, including their occurrence and seasonality, is crucial for public health and can inform preventive measures. To properly understand and address the spread of these viruses, it is crucial to implement programs based on One Health principles. The One Health is an integrative, collaborative strategy that recognizes the interconnectedness of human, animal, and environmental health. This holistic framework serves to address complex health issues through a multidisciplinary approach that includes human medicine, veterinary science, and environmental studies—particularly entomology in relation to vector-borne diseases. This aspect is crucial in understanding the dynamics of vector-borne diseases within ecosystems shared by humans and animals. For instance, outbreaks of diseases that have zoonotic origins involve both wildlife reservoirs and vector species, such as the WNV. A survey of viruses and mosquito surveillance can benefit the One Health approach as well because it allows detection before infection of humans and horses [43]. An integrated One Health surveillance system that monitors mosquitoes and birds across multiple European countries has already proven effective in the early detection of WNV circulation, with an example of Italy as a model for organizing an integrated network to monitor WNV [44]. This integration enhances not only the capacity for early detection but also facilitates timely response measures crucial for safeguarding public health across multiple domains. The development of the One Health Framework by the European Centre of Disease Prevention underlines the need to implement this approach in wider, cross-border areas [45]. Building on previous achievements in the field, One Health has expanded its focus to include a wider array of issues, such as food security and safety, vector-borne diseases, climate variability, and antimicrobial resistance [46]. Since 2011, Croatia has taken a step in the right direction regarding the One Health approach by introducing passive flavivirus surveillance and establishing the CRONEUROARBO network, which yielded important data on the circulation of viruses in humans, domestic and wild animals, and vectors. This study plays a part in this approach, aiming to get the complete status of WNV circulation in Croatia.

In 2023, 9 EU/EEA countries reported 709 locally acquired human cases of WNV infection. The season marked the highest number of reported locally infected cases since the peak years of 2018 and 2022 [47]. In Croatia, climate change shows up as milder winters and warmer springs, and 2023 had an average air temperature up to 2.0 °C higher than the historical average [48]. Since spring temperatures are crucial for shaping WNV epidemiology and warmer conditions might amplify virus transmission [49], more WNV is likely to circulate. According to the latest ECDC epidemiological summary, since the beginning of 2024 and as of 18 September 2024, 16 countries in Europe reported human cases of West Nile virus infection: Albania, Austria, Bulgaria, Croatia, France, Germany, Greece, Hungary, Italy, Kosovo*, North Macedonia, Romania, Serbia, Slovenia, Spain, and Türkiye, with the Croatian region of Osijek-Baranja county as the latest affected [50].

In Europe, several lineages of the WNV have been identified, with lineages 1a and 2 being the primary strains associated with human infections. Still, since the emergence of lineage 2 in the early 2000s, the latter has become the predominant [51]. As mentioned before, the expansion of the WNV in temperate regions is broadly associated with this shift. More specifically, lineage 2 accounts for 82% of all WNV sequences detected in Europe so far, found in 15 European countries [52]. Although Croatia’s first records of WNV infections were recorded in the 1970s, the first human clinical cases of WNV neuroinvasive infection were detected in 2012 when molecular characterization revealed WNV lineage 2 [11]; the next year, which was the second subsequent year of the WNV outbreak, molecular analyses also confirmed infections with lineage 2 [53]. This lineage was again confirmed in patients with WNND in 2017 and 2018, as well as in two dead goshawks (*Accipiter gentilis*) in 2018 [12].

The phylogenetic analysis of this study’s results suggests the Hungarian origin of our strain, and this is supported by several aspects. According to a comprehensive study focusing on the evolutionary dynamics of WNV, Hungary represents an important ecological niche for the virus, which is primarily linked to the route of migratory birds. This fact suggests that the transition of the virus into the Balkan countries happens primarily throughout Hungary [54]. A further comprehensive study compared flight data from several migratory bird species with the number of WNV cases within Europe and performed a time-calibrated phylodynamic analysis using the available complete WNV genomes. The results identified Austria and Hungary as the two main centers of the spread of the virus and demonstrated a close genetic relationship between the European and South African WNV strains, which can be primarily linked to the movement of birds along the African European route, further supporting our results [55]. Moreover, results from neighboring countries further strengthen theories regarding the potential Hungarian origin of the WNV strain identified in this study. According to a cross-border study conducted in Hungary and Austria, where wild birds, horses, and human samples were tested for the presence of WNV by RT-PCR and serological methods, the phylogenetic analysis of identified genomes grouped in a separate clade and showed a close genetic relationship with a Hungarian WNV strain from 2004 [56]. Results of a Slovenian study examining the WNV situation in the country revealed a close genetic relationship of identified WNV strains with a Hungarian and Austrian Czech sequence, respectively [57]. Two independent studies from Serbia revealed a close genetic relationship between Serbian and Hungarian WNV strains [58,59]. Birds have a crucial role in the life cycle of WNV as they serve as the primary reservoir for the virus, with residential birds maintaining the virus in endemic areas while migratory birds play a role in virus introduction/spread into new areas [60].

This study revealed the presence of WNV in *Cx. pipiens* mosquitoes in Croatia. Although in the case of samples from Osijek-Baranja county, the virus could not be identified, it is assumed to be a WNV infection based on the presence of *Cx. pipiens* mosquitoes in the sample, and that the reported human case from the area was WNND. More importantly, this early detection occurred a month before confirming the first human case mentioned above. The second positive pool was from Vukovar-Srijem county, which also contained *Cx. pipiens*, and sequencing of those amplicons revealed WNV lineage 2 partial NS5 gene sequences. These results present the first detection and sequence of WNV in mosquitoes in Croatia and confirm that human WNND cases in this region are infections transmitted by the local *Culex* mosquitoes and lineage 2 causing the infections.

## 5. Conclusions

Currently, no measures are implemented in Croatia to control the spread of WNV until human WNND cases are reported, and depending on the number of cases, epidemiological measures are advised. This is something that should be improved in the future. In this instance, the One Health framework could be useful because it supports comprehensive strategies aimed at enhancing our understanding of how interconnected factors influence disease transmission among humans, animals, and vectors within shared environments [45]. Continued research efforts under this paradigm are crucial for effective public health interventions. Surveillance of mosquitoes and arboviruses within this framework is important but often requires significant resources and capacities. To maintain it, some new methods for its optimization should be involved. The use of Box Gravid Mosquito traps coupled with honey-baited FTA cards could be an effective way to detect the circulation of mosquito-borne viruses more rapidly [61]. Another approach is to implement a rapid diagnostic system for WNV that can be used under field conditions, such as a portable qRT-PCR approach [34] or a lateral flow microarray immunoassay [62]. These kinds of methods should be further tested in the field and implemented to achieve timely prevention and control of WNV.

The identification of WNV lineage 2 in mosquitoes has now completed the roles in the infection cycle for this virus within continental Croatia. This particular strain is recognized as highly infectious, presenting considerable risks to both human and animal health. Confirmed cases of infection in both humans and animals, especially birds, are evidence of the circulation of WNV in this area. In addition, Croatia is on migratory paths of birds coming from areas that are the main centers of the spread of the virus—Austria and Hungary, which is a reason for caution. It is crucial to implement preventive strategies against potential infections and outbreaks by utilizing our current understanding of the virus, its reservoirs, and its vectors. The One Health approach is instrumental in facilitating these preventive measures, integrating the surveillance of mosquitoes to enable the early detection and management of arbovirus transmission dynamics.

This article is a revised and expanded version of a presentation entitled “First detection of West Nile Virus RNA in field-collected mosquitoes in Croatia”, which was presented at the 23rd European Society of Vector Ecology Conference, Montpellier, France, 14 to 17 October 2024 [63].

## Figures and Tables

**Table 1:** Oligonucleotide primers used for *Flavivirus* detection.

Primer	Sequence (5′–3′) ^1^
cFD2	GTGTCCCAGCCGGCGGTGTCATCAGCG
MAMD	AACATGATGGGRAARAGRGARAA
FS778	AARGGHAGYMCDGCHATHTGGT
WN10533-10552	AAGTTGAGTAGACGGTGCTG
WN10625-10606	AGACGGTTCTGAGGGCTTAC
WN10560-10579	CTCAACCCCAGGAGGACTGG

^1^ Probes are labeled with the fluorescent FAM dye at the 5′ end and with the BHQ1 and TAMRA dyes at the 3′ end.

## Data Availability

The genome sequence presented in this study is openly available in the GenBank database Acc.No.: PQ468649.
